# Sulphated Flavonoids: Biosynthesis, Structures, and Biological Activities

**DOI:** 10.3390/molecules23020480

**Published:** 2018-02-23

**Authors:** Yanna C. F. Teles, Maria Sallett R. Souza, Maria de Fátima Vanderlei de Souza

**Affiliations:** 1 Department of Chemistry and Physics, Agrarian Sciences Center, Universidade Federal da Paraíba, Areia 58397-000, PB, Brazil; yanna@cca.ufpb.br; 2 Post graduation Program in Bioactive Natural and Synthetic Products, Health Sciences Center, Universidade Federal da Paraíba, João Pessoa 58051-900, PB, Brazil; sallett@gmail.com; 3 Post graduation in Development and Technological Innovation in Medicines, Health Sciences Center, Universidade Federal da Paraíba, João Pessoa 58051-900, PB, Brazil

**Keywords:** sulphated flavonoids, sulphation, secondary metabolism, sulphotransferases

## Abstract

The great diversity of enzymatic reactions in plant secondary metabolism allows the continuous discovery of new natural compounds and derivatives. Flavonoids, for example, can be found as aglycone or as several sorts of glycosylated, acetylated, methylated, and sulphated derivatives. This review focuses on sulphated flavonoids, an uncommon group of flavonoid derivatives found in some plant families. This work presents a compilation of sulphated flavonoids and their natural sources reported in the literature. Biosynthetic aspects and biological activities have also been reviewed, showing that these particular kinds of natural compounds play an interesting role in plant metabolism, as well as being potential candidates for the development of new drugs.

## 1. Introduction

Secondary metabolites are specialized compounds produced by plants and microorganisms in response to environmental changes as a result of the organism adapting to, or as a defence mechanism against, pathogens or predators. They are not involved in the central (primary) metabolism and their biosynthesis is frequently taxonomically restricted. Some examples of secondary compounds are alkaloids, terpenoids, lignans, and phenolic compounds [1].

The great diversity of secondary metabolism in plants has given phytochemistry research a continuous process of discovery of new naturally occurring compounds and derivatives. The production of over 200,000 structures from plant metabolism is estimated, and a great part of this number may be due to enzymatic modifications of known compounds resulting in derivatives, such as prenylated, acetylated, methylated, sulphated, glucuronated, and glycosylated substances, among others [2,3].

**Figure 1:**
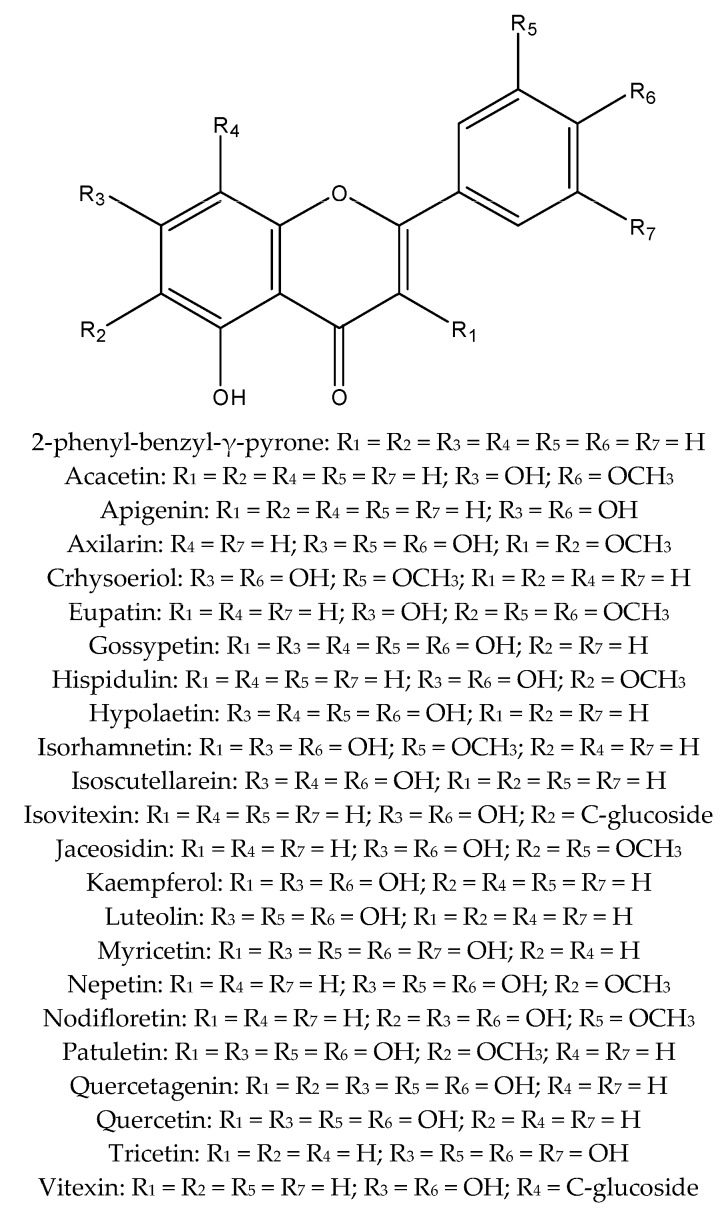
Flavonoid characteristic skeleton and known flavonoids.

Polyphenols are one of the most relevant groups of secondary metabolites. They are usually divided in classes, such as stilbenes, phenolic acids and simple derivatives, lignans, tannins, and flavonoids. Flavonoids comprise the most studied group of polyphenols [4]. They are characterized by the 2-phenyl-benzyl-γ-pyrone nucleus, with great structural diversity resulting in different flavonoid types, e.g., aurones, flavanonols, isoflavones, flavones, flavonols and anthocyanins. The structural variation is also a result of different attached substituents. For example, flavonoids can suffer enzymatic methylation by methyltransferases and glycosylation by specific glycosyltransferases [5]. Figure 1 shows the flavonoid basic skeleton and known flavonoids from which sulphated derivatives have been identified.

The biological and pharmacological activities of flavonoids have been described. They are known to protect plants from biotic and abiotic stresses, they can absorb UV-light and possess antioxidant potential [6]. In human health, many flavonoids have been related to prevention of cardiovascular disease, ischemia, and inflammation, among others. Quercetin, for example, has been shown to inhibit ATP hydrolysis and synthesis by the ATP synthase [7,8].

**Figure 2:**
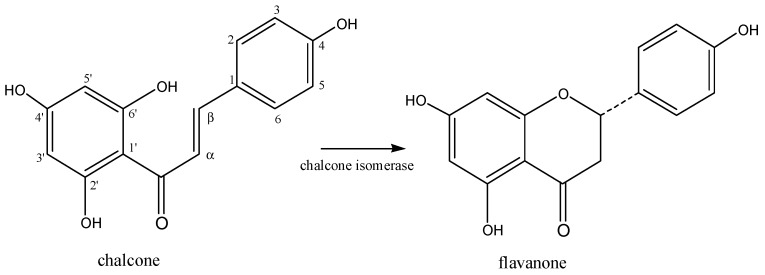
Chalcone isomerization to form a flavanone.

Flavonoid biosynthesis is part of the phenylpropanoid pathway, and starts with the condensation of one *p*-coumaroyl-CoA molecule with three malonyl-CoA molecules (biosynthesis by mixed pathway) resulting in a chalcone (4,2′,4′,6′-tetrahydroxychalcone). This chalcone is isomerized by chalcone isomerase to form a flavanone [5] (Figure 2).

Flavanone suffers additional enzymatic reactions to yield several different flavonoid sub-classes or types, e.g., flavones, flavonols, flavanonols (dihydroflavonols), isoflavones, and anthocyanins [5].

Under natural conditions, flavonoids can be found as aglycones or as several sorts of glycosides, prenylated, acetylated, methylated, and sulphated derivatives. Each different substituent or pattern of substitution creates new derivatives with particular characteristics and properties [9].

This review will focus on sulphated flavonoids, an uncommon group of flavonoid derivatives found in some plant families, that has developed pharmaceutical interest especially because of their potential use as candidates in the development of new drugs.

## 2. Methodology

The present study was carried out based on a literature review using the keyword ‘sulphated flavonoids’. The scientific publications were selected focusing on sulphated flavonoids with a sulphate group attached to the aglycone, considering their chemical structures, biosynthesis, and biological activities. The SciFinder database (Chemical Abstracts Service, Columbus, OH, USA) has been used. The literature source and the selected studies referred to in this review have been published in the English language.

## 3. Sulphated Flavonoids: General Information and Chemotaxonomic Aspects

Sulphated flavonoids may be the less common flavonoid derivative compounds found in some specific plant families [10]. They can be single sulphate or multi-sulphate esters of known flavonoid skeletons, usually flavones or flavonols [9,10,11,12]. The sulphate group is negatively charged, and the counter ion is usually not described although some authors have reported potassium and sodium as counter ions [13].

**Figure 3:**
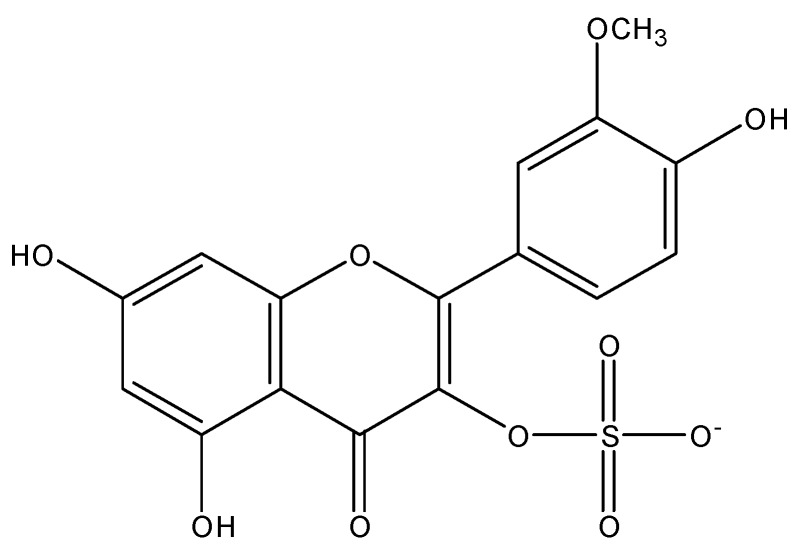
Structure of Persicarin, the first sulphated flavonoid reported.

The first sulphated flavonoid was reported in 1937, isorhamnetin 3-sulphate (persicarin), isolated from *Polygonum hydropiper* L. (Polygonaceae) (Figure 3) [14]. Later, other related flavonoids were isolated, usually from plants found in swampy areas. In fact, a strong correlation has been shown between plants growing near aquatic areas rich in mineral salts and the biosynthesis of sulphated flavonoids, being considered an ecological adaptation to the environment. It is estimated that 150 sulphated flavonoids of natural occurrence have been identified, including those with the sulphate attached to sugars [13].

Sulphate flavonoids are found in Angiosperms and have been identified in eudicotyledon and monocotyledon plants. Among monocotyledons, the families Arecaceae (Palmae), Juncaceae, and Gramineae seem to have a greater occurrence of these compounds [10]. The main eudicotyledon representative families are Asteraceae (Compositae), Bixaceae, Malvaceae, Dilleniaceae, *Umbelliferae, and* Verbenaceae [9]. Most of these families are very far from each other in taxonomic terms, indicating no significance in systematic development. The prevalence of these compounds has been shown in certain genera, for example, a survey of over 250 representative taxa in the *Umbelliferae* has shown that *sulphates* occur in three genera, Ammi, Daucus, and Oenanthe [10].

The functional role of flavonoid sulphates in plant cells and tissues is still not clear. They seem to have an important role in co-pigmentation by forming stable complexes with anthocyanin pigments. They also seem to act in regulating plant growth by affecting auxin transport. It has been shown that quercetin 3-sulphate acts as a quercetin antagonist. The sulphated flavonoid reverts the auxin efflux inhibition caused by quercetin. Thus, quercetin 3-sulphate would stimulate auxin transport from the apical tissues [15]. In the plant kingdom, other biological functions have been discovered, including molecular recognition, detoxification, and signalling pathways [9,10].

Flavonoids are known for their great variety of biological activities and several studies have demonstrated pharmacological properties for sulphated flavonoids, *highlighting* their anticoagulant, anti-inflammatory, and antitumor activities [7,8,9,10,11]. The most relevant aspects of biosynthesis, chemical structures, and biological activity will be reviewed in the next sections.

### 3.1. Biosynthesis

Sulphur (S) is considered an essential nutrient for vegetable growth and development. In spite of its relevance, S is present naturally in few organic compounds as amino acids cysteine (Cys) and methionine (Met), proteins, co-enzymes, vitamins, and secondary metabolites such as glucosinolates and sulphoflavonoids. The S metabolism in plants is still poorly understood, although the role of secondary metabolites and certain S-containing peptides, for example, *glutathione,* has been demonstrated to be very important to cell metabolism and plants’ biotic and abiotic interactions [16].

In general, plants assimilate S as sulphate from the soil, where usually its concentration is low. Thus, this uptake usually requires active transporters in roots, phloem, tonoplast and plastid to ensure the S uptake and distribution. The transporters have been shown to play a key role to maintain the homeostasis of S and derived compounds [16,17,18].

Taken up from the soil, ATP-sulphurylase catalyses sulphate assimilation into adenosine-5′-phosphosulphate (APS), followed by reduction into sulphite and sulphide. The sulphide is then used to Cys biosynthesis, by incorporation on the amino acid skeleton of O-acetylserine. APS can also be phosphorylated to 3′-phosphoadenosine-5′-phosphosulphate (PAPS), and then be used for further sulphation reactions in secondary metabolism [18,19].

PAPS required for the sulphation in secondary metabolism is produced in plastids and then exported into the cytoplasm. Thus, cytosolic *sulphotransferases* (SOTs) are able to catalyse the production of sulphated flavonoids and other sulphated secondary metabolites from PAPS sulphate and specific precursors [14]. The SOTs are enzyme isoforms, which act by transferring the functional sulphur group from PAPS to hydroxylated substrates, e.g., flavonoids and other phenolics [9]. Different SOT isoforms are found in the Golgi, where they add sulphate to proteins and carbohydrates that will be sent from the cell [20].

The SOTs seem to act by a flavonoid position-specific mechanism. It has been shown that different SOTs enzymes exhibited specificity for certain hydroxyl positions and aglycones. For example, flavonol SOTs from *Arabidopsis* thaliana show better *affinity* with kaempferol or flavonol glycosides, transferring the sulphate group to a hydroxyl at the 3 or 7 positions. However, flavonol SOTs from Flaveria bidentis produce 4′ and 3′ sulphate derivatives and show more *affinity* with quercetin [21,22].

SOTs in animals have been well studied and the sulphation is a well-known biotransformation reaction suffered by flavonoids and other phenolics. The produced sulphated flavonoids are more polar metabolites and can be easily transported in blood or excreted in urine. Despite the clear role of flavonoid sulphation in animal metabolism, few plant SOTs have been completely studied and further studies are needed to better clarify their substrate specificity, mechanism, and function of sulphation [21,22,23].

### 3.2. Chemical Structures

Sulphated flavonoids are single sulphate or multi-sulphate esters of known flavonoids (Figure 1) and most of them are based on flavones or flavonols [9,10,11,12]. The negatively-charged sulphate group increases the flavonoid polarity (Figure 2). The fact that the sulphated flavonoids are more polar than their respective glucosides and aglycones allows the researchers to separate those flavonoid types by choosing the proper stationary phase and solvent system. The isolation of sulphated flavonoids using Sephadex LH-20, silica gel, C18 silica, Amberlite XAD-7, HPLC C18 column, HPLC C8 column, microcrystalline cellulose TLC, and electrophoresis have been reported [9,18,24,25].

The flavonoid sulphate ester bonds are quite unstable, and this may be the reason why the sulphated flavonoids are not easily identified. They can suffer hydrolysis during the extraction, purification and storage, especially in acid conditions. Enerstvedt et al. (2016) investigated the stability of sulphated flavonoids from *Zostera marina* extract, showing that the compounds presented good stability in solutions containing 0.1 to 1.0% formic acid and in 0.1% trifluoroacetic acid (TFA). However, in the extract containing 0.5% TFA, the sulphated flavones suffered acid hydrolysis decomposing to their corresponding aglycones [17].

It has been reported that the most common flavonoid sulphate esters are represented by sulphation at positions 7 > 3′ > 4′ > 6 > 8 and 3 > 7 > 4′ > 3′, for flavones and flavonols, respectively. However, recently it has been showed that SOTs possess more affinity to specific aglycones and that the order of sulphation may change by previous sulphation. Thus, this sulphation position priority may change according to the flavonoid structure [26].

It is estimated that 150 sulphated flavonoids of natural occurrence have been identified, but this number includes those with sulphate attached to sugars [18]. In this study, we focused on the flavonoids with sulphate attached to the flavonoid nucleus produced by *cytoplasmic* SOTs. The structures of the naturally-occurring sulphate flavonoids, their natural source and references are showed in Table 1.

Most of the sulphated flavonoids have been identified by NMR and the attached sulphate group causes a characteristic chemical shift in neighbouring carbons when compared with the non-sulphated analogues. For example, the sulpho-flavonoid yannin (8-*o*-sulphate isoscutellarein) isolated from Wissadula periplocifolia possesses a sulphate group attached to the hydroxyl at the 8 position of an isoscutellarein aglycone. When comparing the ^13^C NMR data obtained from the same solvent and equipment, it was observed that the carbon at the 8 position was 4 ppm shielded while C-7, C-9 and C-5 were about 4 ppm deshielded [9]. The same pattern of chemical shifts in neighbour carbons and hydrogens has been observed in several other sulphated flavonoids. In general, we observe that the carbon directly attached to the sulphate ester and the carbons in the meta position are shifted *upfield, while protons and carbons in ortho and para positions to the sulphate group* are shifted *downfield*. The structures should be confirmed by mass spectra, especially those with more than one sulphate ester, that can have this *protection and deprotection* pattern annulled by addition effects of multiple sulphate substituents [17].

The concentration of sulpho-flavonoids in plant extracts remains little studied. Dantuluri et al. (2004) applied and optimized the conditions of capillary electrophoresis to analyse complex mixtures of sulphated flavonoids. The developed method was able of separating with reasonable resolution the peaks of quercetin 3,5,7,3′,4′sulphate, apigenin 7,5,4′sulphate, (−)epicatechin 3,5,7,3′,4′sulphate and (+)-catechin 3,5,7,4′,5′sulphate, showing that the technique can be useful to quantify sulphated flavonoids in plant samples [25]. In other study, HPLC combined with diode array detection (DAD) was used for quantitative determination of sulphated flavonoids in samples of *Zostera noltii* collected in two different geographical zones*.* One of the samples was found to be dominated by apigenin 7-sulfate (up to 83% of the total flavonoids), whereas the other one was characterized by diosmetin 7-sulfate (up to 93% of the total flavonoids). Thus, based on sulphated flavonoids amount the authors found evidences of chemotypes within the species *Z. nolti* [42].

### 3.3. Biological Activities

Flavonoids are well known for their many beneficial biological and pharmacological functions. Structural modifications, for example, sulphation, methylation, and glycosylation usually change their solubility, stability, and biological activities [4]. The negatively-charged sulphated derivatives have greater water solubility, and the negative charge is very important in interactions with biological targets [43].

Several biological activities have been investigated for sulphated flavonoids, such as anticoagulant, antiplatelet aggregation, anti-inflammatory, immunomodulatory, and antitumor effects [9,43]. Among them, the anticoagulant and antiplatelet aggregation activities are well studied. Heparin, a naturally-occurring anticoagulant, is a negatively-charged sulphated polysaccharide and the negative charge of sulphated flavonoids seems to allow them to bind to heparin receptors [43,44]. The anticoagulant potential of persicarin (isorhamnetin 3-sulphate) and isorhamnetin were evaluated by Ku et al. (2013). The anticoagulant effect of persicarin was greater than that of isorhamnetin, suggesting that the sulphate group of persicarin regulates its anticoagulant action [45]. The semi-synthesized quercetin derivative, disodium quercetin-7,4′-disulphate, was able to inhibit pig platelet aggregation induced by thrombin [46]. Quercetin 3,7,3′,4′-tetrasulphate and quercetin 3-acetyl-7,3′,4′-trisulphate have demonstrated anticoagulant and antiplatelet properties, to prolong activated partial thromboplastin and prothrombin clotting times [11,47]. Other synthesized flavonoids, such as (–)-epicatechin persulphate, (+)-catechin persulphate, quercetin persulphate, morin persulphate, 2′,3,3′,6-tetrahydroxyflavonol persulphate have been described as anticoagulant agents [43].

The antimicrobial effects of sulphated flavonoids have been investigated. Quercetin 7,3′-dimethyl ether 3-sulphate and kaempferol 7-methyl ether 3-sulphate isolated from *Argyreia speciose* presented MIC of 25.00 µg/mL against *Mycobacterium tuberculosis,* showing a synergistic effect with isoniazid and rifampicin [36]. Myricetin 3′-sulphate showed significant activity against *Trypanosoma brucei* with an IC_50_ value of 8.52 μg/mL [29].

There is a strong interest in understanding the antioxidant and anti-inflammatory potential of sulphated flavonoids because this is one of the main metabolites found in human blood after administration of aglycones. [48,49,50]. Usually, flavonoids with a catechol moiety (*ortho*-dihydroxy) in rings A or B, with a double bond at C2–C3 are COX-2 inhibitors. Pascual-Teresa et al. (2004) showed that quercetin 3′-sulphate downregulates COX-2 expression in human lymphocytes in a dose-dependent way [51].

Sulphated flavonoids have shown antioxidant activity with the ability to scavenge superoxide induced by xanthine–xanthine oxidase. The antioxidant activity of sulphated and non-sulphated flavonoids was evaluated with the 2,2-diphenyl-1-picrylhydrazyl (DPPH) assay. The study compared quercetin vs. quercetin 3′-sulphate and quercetin 3,3′-disulphate. The assay showed that when the hydroxyl groups are substituted by a sulphate, the antioxidant activity failed dramatically [48].

*Polygonum hydropiper* has been widely studied as a great producer of sulphated flavonoids. Some structures isolated from it had their antioxidant potential determined. Quercetin 3-sulphate and isorhamnetin 3,7-disulphate showed greater antioxidant activity than α-tocopherol, a strong naturally-occurring antioxidant [49]. Sulphated flavonoids from *Polygonum hydropiper* also exhibited a potent inhibitory activity on aldose reductase. This enzyme catalyses the reduction of various aldehydes and the reduction of glucose to sorbitol. The high intracellular concentration of sorbitol leads to hyperosmotic conditions responsible for the loss of clarity in the lens in cataract. Among the tested flavonoids from *P. hydropiper*, isorhamnetin 7-disulphate showed the greater inhibitory activity on lens aldose reductase. In addition, removal of the sulphate group seems to decrease aldose reductase inhibition. The results pointed up that sulphated flavonoids may be effective in preventing cataract formation [50].

Cavallaro et al. (2013) showed the antioxidant activity and acetylcholinesterase (AChE) inhibition of kaempferol 6-methyl ether 3-sulphate. They proposed that the activity observed for the ethanolic extract of *Flaveria bidentis* on Alzheimer’s disease is related to the high content of the sulphated flavonoids. They showed that the loss of the 3-sulphate group led to a weaker AChE inhibition, suggesting that the presence of this group is crucial for the interaction with the enzyme [52].

The cytotoxicity of hispidulin 7-sulphate and luteolin 7-sulphate was compared with that of hispidulin and luteolin in the culture of B16F10 cells and revealed that the sulphated group attenuated the cytotoxicity. The study also showed the anti-melanogenic effects of luteolin 7-sulphate at non-toxic concentrations, showing that the compound can be useful in controlling unwanted skin pigmentation [53]. A mixture of sulphated flavonoids, acacetin 7-sulphate (wissadulin) and isoscutellarein 4′-methyl ether 8-sulphate (caicoine) obtained from *Wissadula periplocifolia* was shown to be active against PC-3M prostate carcinoma cells and UVW glioma *cells* [9].

The studies found in the literature indicate that sulphated flavonoids can be an interesting alternative in the search for new drugs, mainly due to their increased water solubility and the presence of the negative-charge promoting interactions with several biological targets.

## 4. Conclusions

This review focused on sulphated flavonoids, showing a compilation of sulphated structures reported in the literature. In addition, biosynthetic aspects and biological activities have been presented, showing that these uncommon natural compounds are interesting potential candidates for the development of new drugs.

## Figures and Tables

**Table 1:** Sulphated flavonoids reported from plants and their sources (species name and family).

Sulphated Flavonoids	Species Name and Family	Reference
Acacetin 7-sulphate	*Wissadula periplocifolia* (Malvaceae)	[9]
Apigenin 7-sulphate	*Tetracera mandagascariensis* (Dilleniaceae) *Bixa orllana* (Bixaceae)	[12,27,28]
Ampelopsin (dihydromyricetin)-3′-sulphate	*Limonium caspium* (Plumbaginaceae)	[29]
Axillarin 7-sulphate	*Centaurea bracteata* (Asteraceae)	[30]
Chrysoeriol 7-sulphate	*Zostera marina* (Zosteraceae)	[31]
Eupatin 3-sulphate	*Brickellia californica*, *B. laciniata* (Asteraceae)	[31]
Gossypetin 3-sulphate	*Malva sylvestris* (Malvaceae)	[32]
Gossypetin 8-*o*-β-d-glucuronide-3-sulphate	*Malva sylvestris* (Malvaceae)	[32]
Hypoaletin 3′-sulphate	*Malva sylvestris* (Malvaceae)	[33]
Hypoaletin 8-sulphate	*Bixa orllana* (Bixaceae)	[28]
Hypolaetin 3′-methyl ether 8-sulphate	*Wissadula periplocifolia* (Malvaceae)	[9]
Hispidulin 7-sulphate	*Lippia nodiflora* (Verbenaceae)	[34]
Hispidulin 4′-sulphate	*Lippia nodiflora* (Verbenaceae)	[34]
Hispidulin 7,4′-disulphate	*Lippia nodiflora* (Verbenaceae)	[34]
Isoscutellarein 8-sulphate	*Wissadula periplocifolia* (Malvaceae)	[9]
Isorhamnetin 3-sulphate	*Senecio galicus* (Asteraceae); *Polygonum hydropiper* (Polygoniaceae)	[13,14]
Isorhamnetin 7-sulphate	*Frankenia pulverulenta (*Frankeniaceae)	[31]
Isorhamnetin 3,7-disulphate	*Flaveria bidentis* (Asteraceae)	[10,31]
Isorhamnetin 3-glucoronide 7-sulphate	*Frankenia pulverulenta (*Frankeniaceae)	[10,31]
Isorhamnetin 3,7,4′-trisulphate	*Acrotrema uniflorum* (Dilleniaceae)	[31]
Isoscutellarein 4′-methyl ether 7-sulphate	*Wissadula periplocifolia* (Malvaceae)	[9]
Isoscutellarein 4′-methyl ether-8-sulphate	*Wissadula periplocifolia* (Malvaceae)	[9]
Isoscutellarein 7,4′-dimethyl ether 8-sulphate	*Wissadula periplocifolia* (L.), *Sidastrum micranthum* (Malvaceae)	[9,35]
Isovitexina 7-sulphate	*Phoenix roebelenii (*Arecaceae)	[28]
Isorientin 7-sulphate	*Phoenix roebelenii (*Arecaceae)	[28]
Jaceosidin 7-sulphate	*Lippia nodiflora* (Verbenaceae)	[34]
Jaceosidin 7,4′-disulphate	*Lippia nodiflora* (Verbenaceae)	[34]
Kaempferol 3-sulphate	*Dillenia bracteata*, *D. triquetra*, *Schumacheria casteinifolia*, *Tetracera alnifolia*, *T. boiviniana*, *T. breyniana*, *T. costata*, *T. oblongata*, *T. rasiflora*, *T. rutenbergii*, *T. volubilis*, *T. willdenowiana* (Dilleniaceae)	[12,31]
Kaempferol 7-sulphate	*F. pulverulenta (*Frankeniaceae)	[31]
Kaempferol 3,7-disulphate	*Reamuria mucronata*, *R. vermiculata* (Tamaricaceae); *Dillenia bracteata*, *Schumacheria castaneifolia* (Dilleniaceae)	[27,31]
Kaempferol 3,7,4′-trisulphate	*Acrotrema uniflorum* (Dilleniaceae)	[27]
Kaempferol 7-methyl ether 3-sulphate	*Ammi visnaga* (Umbeliferaceae); *Acrotrema uniflorum*, *Tetracera alnifolia*, *T. puggei*, *T. rosiflora*, *T. rutenbergii* (Dilleniaceae); *Argyreia speciosa* (Convolvulaceae)	[12,31,36]
Kaempferol 7,4′-dimethyl ether 3-sulphate	*Tamarix apphyla*, *T. nilotica* (Tamaricaceae)	[30,37]
Kaempferol 6,7,4′-trimethyl ether 3-sulphate	*Brickellia longifolia* (Asteraceae)	[31,36]
Kaempferol 3-glucoronide 7-sulphate	*Frankenia pulverulenta (*Frankeniaceae)	[27]
Kaempferol 3-sulphate 7-*o*-α-arabinopyranoside	*Atriplex hortensis* (Chenopodiaceae)	[24]
Luteolin 7-sulphate	*Tetracera stuhimanniana* (Dilleniaceae) *Bixa orllana (*Bixaceae)	[28,31]
Luteolin 4′-sulphate	*Daucus carota* (Umbelliferae)	[28]
Luteolin 3′-sulphate	*Lachenalia unifolia* (Hyacinthaceae)	[28]
Luteolin 7,3′-disulphate	*Zostera marina* (Zosteraceae)	[28]
Luteolin 7-sulphate 3′-glucoside	*Mascarena verscafeltii* (Arecaceae)	[28]
Luteolin 7-sulphate 3′-rutinoside	*Zostera marina* (Zosteraceae); *Mascarena verschaffeltii*, *Opsiandra maya* (Arecaceae)	[28,30]
Luteolin 4′-methyl ether (diosmetin) 7-sulphate	*Zostera marina*, *Z. nana* (Zosteraceae)	[28]
Luteolin 4′-methyl ether (diosmetin) 7,3′-disulphate	*Lachenalia unifolia* (Hyacinthaceae)	[28]
Luteolin 6-hydroxy 7-sulphate	*Lippia nodiflora* (Verbenaceae)	[34]
Luteolin 6-hydroxy 6-sulphate	*Lippia nodiflora* (Verbenaceae)	[34]
Luteolin 6-hydroxy 6,7-disulphate	*Lippia nodiflora* (Verbenaceae)	[34]
Luteolin 7-sulphate 8-C-glucoside	*Phoenix roebelenii (*Arecaceae)	[28]
Myricetin-3′-sulphate	*Limonium caspium* (Plumbaginaceae)	[28]
(2*S*,3*S*)-5-Methyldihydromyricetin-3′-sulphate β-d-glucopyranoside	*Limonium caspium* (Plumbaginaceae)	[28]
(2*S*)-Naringenin 4′-*o*-sulphate	*Tamarix africana* (Tamaricaceae)	[38]
Nepetin 7-sulphate	*Lippia nodiflora* (Verbenaceae)	[34]
Nepetin 3′,4′-sulphate	*Lippia nodiflora* (Verbenaceae)	[34]
Nodifloretin 7-sulphate	*Lippia nodiflora* (Verbenaceae)	[34]
Nodifloretin 6,7-disulphate	*Lippia nodiflora* (Verbenaceae)	[34]
Patuletin 3-sulphate	*Brickellia californica* (Asteraceae)	[10]
Patuletin 7-sulphate	*Lasthenia conjugens*, L. *fremontii* (Asteraceae)	[28]
Patuletin 7-sulphate 3-Glucoside	*Lasthenia conjugens*, L. *fremontii* (Asteraceae)	[28]
Quercetin 3-sulphate	*Acrotrema uniflorum*, *Dillenia bracteata*, *D. triquetra*, *Schumacheria angustifólia*, *S. casteinifolia*, *Tetracera alnifolia*, *T. boiviniana*, *T. breyniana*, *T. costata*, *T. madagascariensis*, *T. masuiana*, *T. oblongata*, *T. rasiflora*, *T. rutenbergii*, *T. sarmentosa*, *T. sellowiana*, *T. tigara*, *T. volubilis*, *T. willdenowiana* (Dilleniaceae); *Hypericum elodes* (Guttiferae); *Oenanthe crocata* (Umbelliferae)	[12,28,34,39]
Quercetin 3,4′-disulphate	*Flaveria bidentis* (Asteraceae)	[13]
Quercetin 3,7-disulphate	*Flaveria bidentis* (Asteraceae)	[13]
Quercetin 3,7,4′-trisulphate	*Flaveria bidentis* (Asteraceae)	[13]
Quercetin 3,7,3′-trisulphate	*Flaveria bidentis* (Asteraceae)	[13]
Quercetin 3,3′,4′,7-tetrasulphate	*Flaveria bidentis* (Asteraceae)	[40]
Quercetin 7-methyl ether 3-sulphate	*Ammi visnaga* (Umbeliferaceae)	[31]
Quercetin 4′-methyl ether 3,7-disulphate	*Reaumuria vermiculata* (Tamaricaceae)	[37]
Quercetin 7-methyl ether 3,5,4′-trisulphate	*Tamarix apphyla* (Tamaricaceae)	[37]
Quercetin 7,4′-dimethyl ether 3-sulphate	*Flaveria chloraefolia* (Asteraceae)	[41]
Quercetin 7,3′dimethyl ether 3-sulphate	*Argyreia speciosa* (Convolvulaceae)	[36]
Quercetin 7,4′-dimethyl ether 3,3′-disulphate	*Acrotrema uniflorum* (Dilleniaceae)	[10]
Quercetin 3-acetyl-7,3′,4′-trisulphate	*Flaveria bidentis* (Asteraceae)	[11]
Quercetin 3-sulphate 7-o-α-arabinopyranoside	*Atriplex hortensis* (Chenopodiaceae)	[24]
Quercetagetin 3-methyl ether 7-sulphate	*Neuroleana oaxacana* (Asteraceae)	[13]
Quercetagetin 6,7-dimethyl ether 3-sulphate	*Brickellia veronikaefolia* (Asteraceae)	[13]
Quercetagetin 6,7,3′-trimethyl ether 3-sulphate	*Brickellia californica* (Asteraceae)	[13]
Quercetagetin 6,7,4′-trimethyl ether 3-sulphate	*Brickellia longifolia* (Asteraceae)	[13]
Tricetin 3′-sulphate	*Lachenalia unifolia* (Hyacinthaceae)	[28]
Tricetin 7,3′-disulphate	*Lachenalia unifolia* (Hyacinthaceae)	[28]
(2*S*,4*R*)-5,7,4′-Trihydroxyflavan-4-ol 5,7-disulphate	*Tamarix africana* (Tamaricaceae)	[38]
(2*S*)-5,7,4′-Trihydroxyflavan 7-*o*-sulphate	*Tamarix africana* (Tamaricaceae)	[38]
Vitexina 7-sulphate	*Washingtonia robusta (*Arecaceae)	[28]
